# Supplementary material for: Co-existence of synaptic plasticity and metastable dynamics in a spiking model of cortical circuits
Source: bioRxiv. 2024 May 9:2023.12.07.570692. Originally published 2023 Dec 8. Preprint. [Version 2] doi: 10.1101/2023.12.07.570692 (PMC10723399; doi:10.1101/2023.12.07.570692)
Supplement: Supplementary file 1 [file NIHPP2023.12.07.570692v2-supplement-1.pdf]

bioRxiv preprint doi: https://doi.org/10.1101/2023.12.07.570692; this version posted May 9, 2024. The copyright holder for this preprint (which was not certified by peer review) is the author/funder, who has granted bioRxiv a license to display the preprint in perpetuity. It is made available under aCC-BY 4.0 International license.

A Supplementary Figures

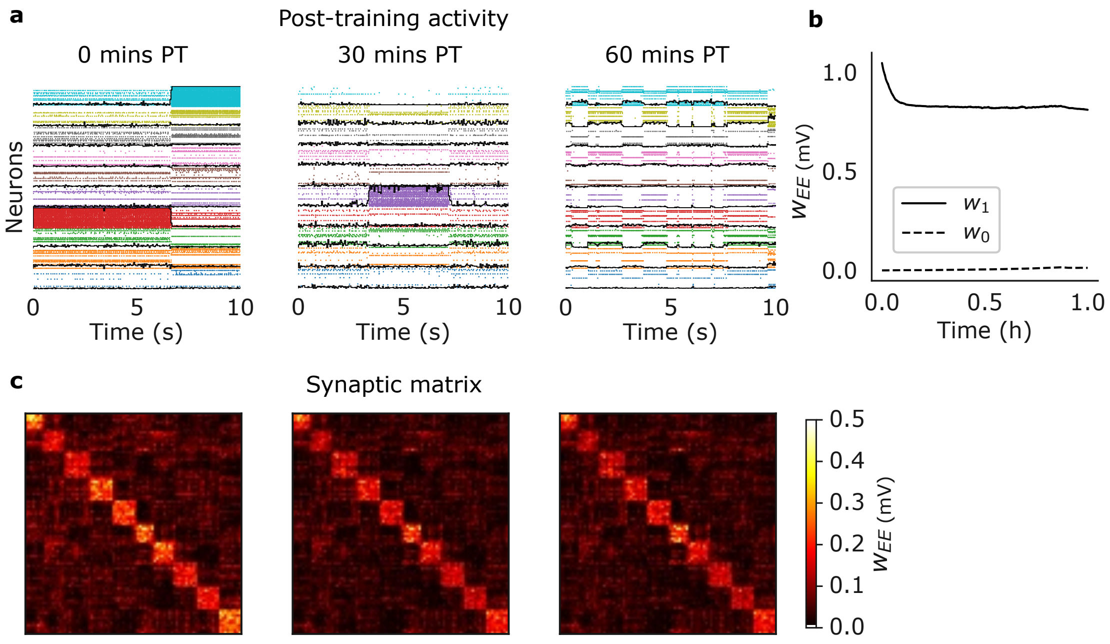

Figure S1: Results from training the basic network of Fig. 2 with $\beta = 0$ in Eq. 1. (a) Rasterplot of excitatory neurons taken immediately after training (left), 30 minutes after training (middle), and 60 minutes after training (right). Same keys as Fig. 2 of the main text. (b) Averaged post-training excitatory synaptic weights as a function of time. w_1: mean weights across synapses connecting neurons sharing at least one stimulus; w_0: mean weights across synapses connecting neurons sharing no stimuli. (c) Synaptic matrix of the network at the same times as in (a) showing the formation of clusters from the block structure of the matrix.

bioRxiv preprint doi: https://doi.org/10.1101/2023.12.07.570692; this version posted May 9, 2024. The copyright holder for this preprint (which was not certified by peer review) is the author/funder, who has granted bioRxiv a license to display the preprint in perpetuity. It is made available under aCC-BY 4.0 International license.

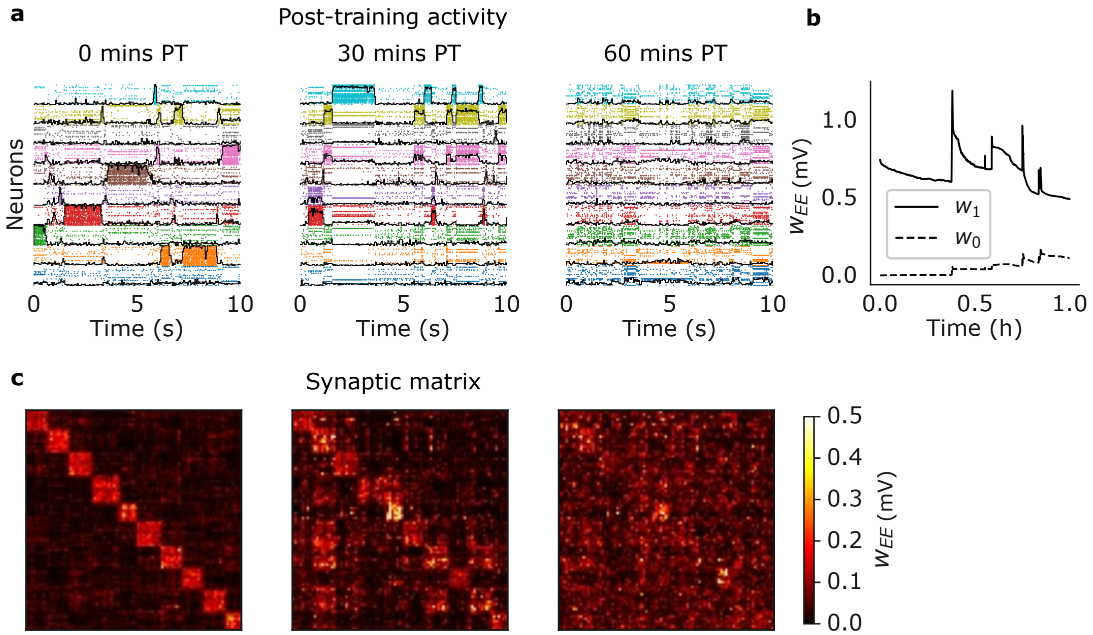

Figure S2: Results from training the basic network of Fig. 2 with $\gamma = 0$ in Eq. 2. Same keys as Fig. S1.

bioRxiv preprint doi: https://doi.org/10.1101/2023.12.07.570692; this version posted May 9, 2024. The copyright holder for this preprint (which was not certified by peer review) is the author/funder, who has granted bioRxiv a license to display the preprint in perpetuity. It is made available under aCC-BY 4.0 International license.

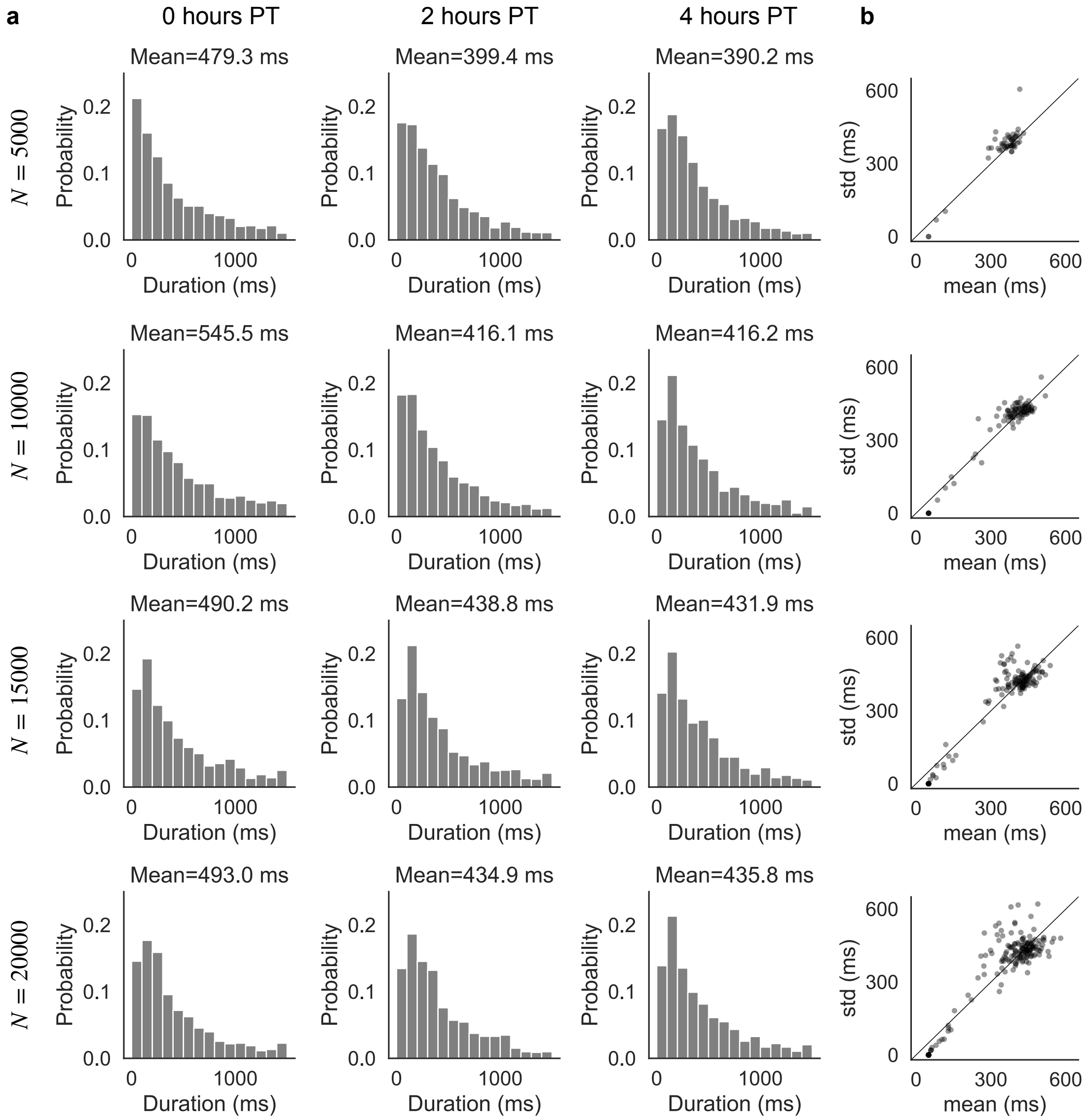

Figure S3: Distributions of durations of cluster activations vs. network size for the networks of Fig. 4. (a) Distributions of durations right after training (left column), 2 hours post-training (middle) and 4 hours post-training (right) for different network sizes. Means tend to decrease with post-training time and increase with network size, approaching stability 4 hours post-training and for $N \geq 15,000$. (b) Scatterplots of standard deviation vs. mean of durations for the corresponding networks in (a), superimposed to the identity line. Each circle corresponds to a cluster. For the majority of the clusters, the standard deviations are approximately equal to the means as expected for an exponential distribution.

bioRxiv preprint doi: https://doi.org/10.1101/2023.12.07.570692; this version posted May 9, 2024. The copyright holder for this preprint (which was not certified by peer review) is the author/funder, who has granted bioRxiv a license to display the preprint in perpetuity. It is made available under aCC-BY 4.0 International license.

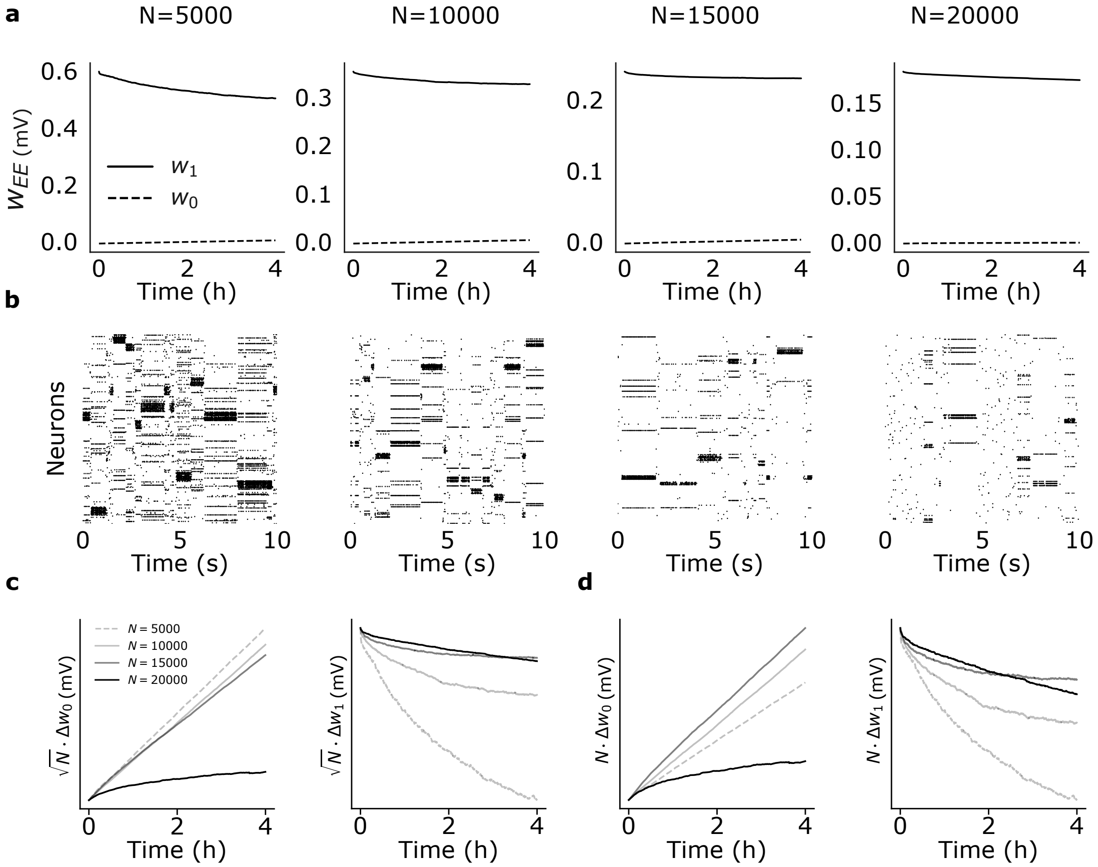

Figure S4: Effect of network size on synaptic dynamics after training. Same as Fig. 4 of the main text but for a different scaling, namely $f \propto 1/\sqrt{N}$, $Q \propto \sqrt{N}$ (see the main text). (a) Time dependence of average synaptic weights w_1 and w_0 after training for networks of different size N, with $N_E = 0.8N$ excitatory neurons. Scaling laws were $f = 1/Q$ with $Q = \sqrt{N/10}$, giving $N_Q = fN_E = 0.8\sqrt{10N}$ neurons in each cluster (where Q and N_Q were rounded to the nearest integer). From left to right, $Q = 22, 32, 39$ and 45. (b) Raster plots of the network's activity 4 hours after training for the corresponding networks in (a). (c) Plots of $\sqrt{N}\Delta w_0$ and $\sqrt{N}\Delta w_1$ vs. time after training for the different network in (a) (note the difference with panel c of Fig. 4 of the main text). Observations were taken 0 to 4 hours post training. (d) Same as (c) for $N\Delta w_{\mathbb{C}}$ vs. time.
